# Astrocyte-Derived Pleiotrophin Mitigates Late-Stage Autoimmune CNS Inflammation

**DOI:** 10.3389/fimmu.2021.800128

**Published:** 2022-01-03

**Authors:** Mathias Linnerbauer, Lena Lößlein, Daniel Farrenkopf, Oliver Vandrey, Thanos Tsaktanis, Ulrike Naumann, Veit Rothhammer

**Affiliations:** Department of Neurology, University Hospital Erlangen, Friedrich–Alexander University Erlangen–Nürnberg, Erlangen, Germany

**Keywords:** astrocytes, multiple sclerosis, autoimmunity, CNS inflammation, protective

## Abstract

Astrocytes are the most abundant glial cells in the central nervous system (CNS) with the capacity to sense and react to injury and inflammatory events. While it has been widely documented that astrocytes can exert tissue-degenerative functions, less is known about their protective and disease-limiting roles. Here, we report the upregulation of pleiotrophin (PTN) by mouse and human astrocytes in multiple sclerosis (MS) and its preclinical model experimental autoimmune encephalomyelitis (EAE). Using CRISPR-Cas9-based genetic perturbation systems, we demonstrate *in vivo* that astrocyte-derived PTN is critical for the recovery phase of EAE and limits chronic CNS inflammation. PTN reduces pro-inflammatory signaling in astrocytes and microglia and promotes neuronal survival following inflammatory challenge. Finally, we show that intranasal administration of PTN during the late phase of EAE successfully reduces disease severity, making it a potential therapeutic candidate for the treatment of progressive MS, for which existing therapies are limited.

## Introduction

Multiple Sclerosis (MS) is a chronic autoimmune disease of the central nervous system (CNS) characterized by acute and chronic waves of inflammation causing demyelination and axonal loss, which clinically manifest as transient as well as accumulating neurological deficits. While our increasing understanding of peripheral immune cell activation and CNS infiltration has led to the development of efficient immune therapies targeting the initial stages of MS, mechanistic insight into the role of CNS-resident cell populations including astrocytes and microglia in chronic disease progression as well as their therapeutic potential have been limited (1, 2). However, based on their strategic location in the CNS and the multitude of interactions with CNS-resident and infiltrating cells, glial cells offer a hitherto neglected potential to target poorly treatable late stages of autoimmune CNS inflammation (3). In this context, recent studies support the notion that in addition to disease-promoting effects, distinct astrocyte subpopulations have the potential to exert protective functions during chronic CNS inflammation (4–8). However, pharmacological induction of these phenotypes and therapeutic exploitation of protective astrocyte-derived mediators is missing.

In this context, we here identify Pleiotrophin (PTN) as a novel astrocyte-derived mediator with anti-inflammatory and neuroprotective functions and potential relevance as druggable factor. PTN is a highly conserved developmentally regulated peptide (9). It is part of the heparin-binding growth factor family of proteins (9) and is predominantly expressed in nervous tissue (10, 11), where it has been described as a growth factor in early embryonic stages (12) as well as a protooncogene (13) with highest expression levels in glioblastoma (9). However, growing evidence suggests that PTN also plays a role in degenerative and inflammatory diseases. Especially its involvement in CNS diseases has attracted increasing research interest. Indeed, PTN is upregulated in Parkinson’s Disease (PD) (14), Alzheimer’s Disease (AD) (15), acute ischemic brain injury (16) and different forms of drug abuse (17–19). However, its relevance in autoimmune CNS inflammation is unclear.

In this study, we investigate the role of astrocyte-derived PTN in the context of neuroinflammation, revealing its anti-inflammatory effects and significance in late stages of experimental autoimmune encephalomyelitis (EAE), an animal model of MS, as well as its therapeutical potential to alleviate late-stage CNS inflammation.

## Results

### PTN Is Expressed in Mouse and Human Astrocyte Subclusters During CNS Inflammation

**Figure 1:**
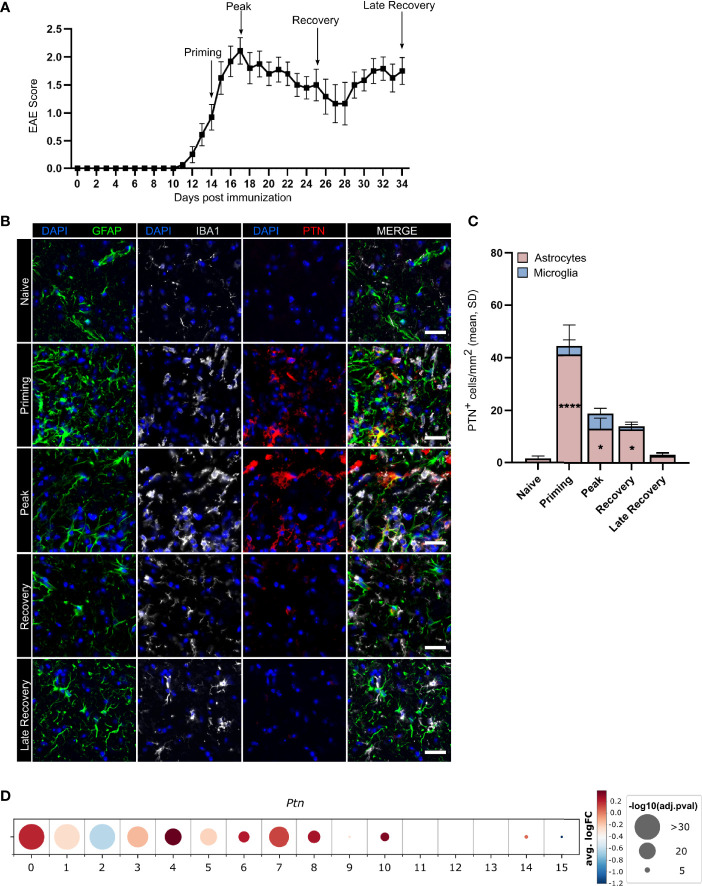
Astrocytes upregulate PTN production during neuroinflammation. **(A)** EAE development in mice used for expression analysis of astrocytic and microglial PTN expression, timepoints analyzed indicated by arrows. Data shown as mean ± S.E.M. *n* = 27. Immunostaining **(B)** and quantification **(C)** of GFAP^+^PTN^+^ astrocytes and IBA1^+^PTN^+^ microglia in lumbar spinal cord across EAE stages. Scale bar represents 100 µm. Quantifications show mean value and individual values of each timepoint. Data shown as mean ± SD. Dunnett’s multiple comparisons test relative to naïve expression *P < 0.05, ****P < 0.0001. **(D)** *PTN* expression by human astrocyte clusters. Data obtained from Wheeler et al. (7).

To investigate PTN regulation in glia cells during autoimmune CNS inflammation, we induced EAE in wild-type C57BL/6J mice by immunization with the myelin oligodendrocyte glycoprotein epitope 35-55 (MOG_35–55_) in complete Freund’s adjuvant (CFA), followed by injection of pertussis toxin, and analyzed PTN immunoreactivity in GFAP^+^ astrocytes and IBA1^+^ microglia in lumbar spinal cord tissue at distinct disease stages (**Figures 1A** **–C**). While microglia upregulated PTN during peak of EAE to a limited extent only, astrocytes demonstrated a significant increase in PTN expression particularly during the priming phase, followed by a reduction to baseline in the late recovery phase (**Figures 1B, C** **)**. These data suggest that PTN is actively regulated in astrocytes but not in microglia in response to an inflammatory micromilieu.

Since numerous studies have recently demonstrated the vast heterogeneity of astrocytes states in inflamed tissue (2, 4, 6, 8), we next investigated whether *Ptn* expression was associated with certain astrocyte subtypes or activation states. Analysis of public transcriptome data of 9,673 cortical and cerebellar astrocytes derived from 20 patients with MS and 28 control individuals demonstrated upregulation of *PTN* in multiple subclusters of MS patients compared to controls (**Figure 1D**). Interestingly, cluster 4, which showed the highest upregulation of *PTN* and was associated to a neurotrophic signature, was reduced 6.2-fold in MS patient samples compared to controls, indicating that there may be a loss of protective astrocyte functions during neuroinflammation (**Supplementary Figures 1A, B** **)**. To further validate our findings in the context of pathogen-mediated inflammation, we analyzed a recently published single cell transcriptome dataset from mouse astrocytes following peripheral stimulation with Lipopolysaccharide (LPS) (4). Indeed, *Ptn* upregulation was detected in clusters 3, 4 and 5, recapitulating our findings in autoimmune inflammation. Moreover, this analysis revealed a spatial localization of *Ptn* in upper and lower cortical layers, which have been described to correlate as layers in which inflammation strongly correlates with disease progression and severity in MS patients (**Supplementary Figures 1C, D** **)** (20).

Collectively, these data suggest that astrocytes may play a key role as cellular source of PTN during neuroinflammatory events both in mice and men.

### Astrocyte-Derived Pleiotrophin Promotes Recovery From Acute CNS Inflammation

**Figure 2:**
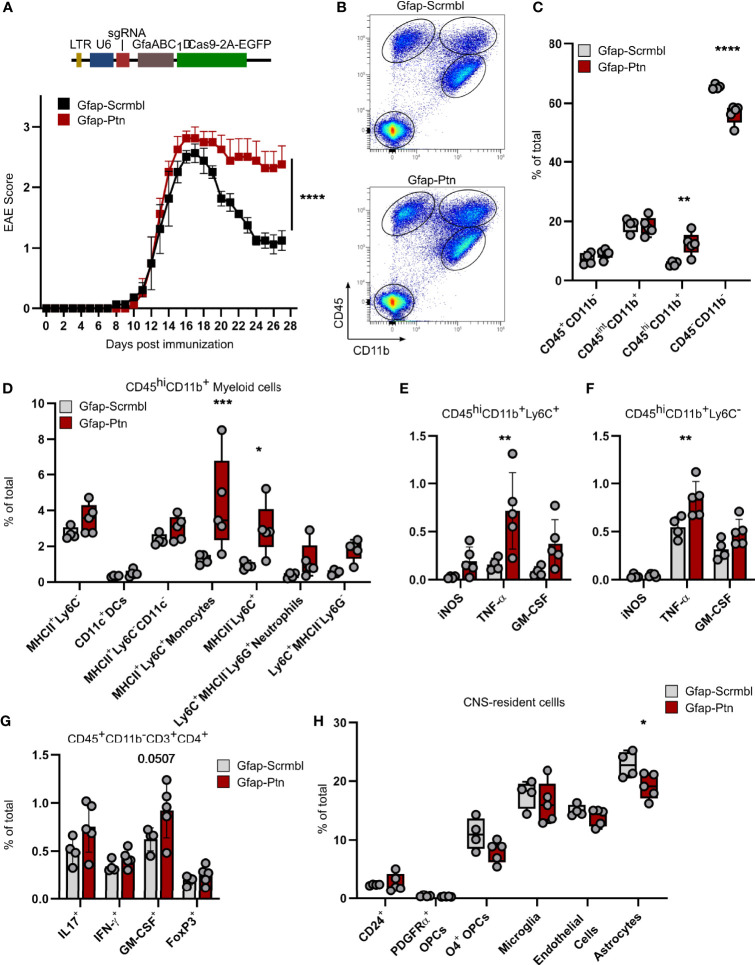
Astrocyte-derived Pleiotrophin is required for recovery from acute neuroinflammation. **(A)** Schematic of lentiviral vector (Top). EAE progression in mice transduced with Gfap-Scrmbl (*n* = 5) or Gfap-Ptn (*n* = 5). Representative of two independent experiments. Data shown as mean ± S.E.M. Two-way repeated measures ANOVA. ****P < 0.0001. **(B)** Representative scatterplots (entire group concatenated) and quantification **(C)** of cellular abundance in the CNS (brain and spinal cord) of Gfap-Scrmbl (black, *n* = 4) and Gfap-Ptn (red, *n* = 5) mice according to CD45 and CD11b expression at day 28 post immunization. Data shown as mean ± SD. Sidak’s multiple comparisons test. **P < 0.01, ****P < 0.0001 **(D)** Abundance of CD45^hi^CD11b^+^ myeloid cells and quantification of their cytokine production **(E, F)** in CNS (brain and spinal cord) of Gfap-Scrmbl (black, n = 4) and Gfap-Ptn (red, *n* = 5) mice at day 28 post immunization. Data shown as mean ± SD. Sidak’s multiple comparisons test. *P < 0.05, ***P < 0.001. **(G)** Abundance of CD3^+^CD4^+^ T cell subsets in CNS (brain and spinal cord) of Gfap-Scrmbl (black, n = 4) and Gfap-Ptn (red, n = 5) mice at day 28 post immunization. Data shown as mean ± SD. Sidak’s multiple comparisons test. **(H)** Abundance of CNS-resident cell populations (brain and spinal cord) in Gfap-Scrmbl (black, *n* = 4) and Gfap-Ptn (red, *n* = 5) mice at day 28 post immunization. Data shown as mean ± SD. *P < 0.05.

To evaluate the effects of astrocyte-derived PTN on the pathogenesis and resolution of CNS inflammation, we used a CRISPR-Cas9 driven approach to inactivate *Ptn* expression in astrocytes. For this, we injected a lentiviral vector coding for *Gfap*-driven CRISPR–Cas9 and *Ptn*-targeting or control sgRNA into the cerebral ventricles of C57BL/6J wild-type mice and induced EAE as described before (6, 7). Knockdown of *Ptn* in astrocytes exacerbated EAE, particularly during late stages, suggesting that astrocyte-derived PTN exerts protective effects and promotes recovery from acute CNS inflammation (**Figure 2A** and **Supplementary Figures 2A, B** **)**. Multicolor flow cytometry of CNS cells at day 27 post immunization, followed by non-parametric, permutation based analysis (21), revealed that inactivation of *Ptn* expression in astrocytes lead to a significant increase in activated CD45^hi^CD11b^+^ myeloid cells, which was predominantly driven by Ly6C^+^MHCII^+^ and Ly6C^+^MHCII^-^ monocyte infiltration (**Figures 2B** **–D**) and accompanied by an increase in activation markers of CNS infiltrating immune cells (**Supplementary Figures 2C–E**). In line with this observation, we detected an increase in TNF-α and GM-CSF produced by Ly6C^+^ and Ly6C^-^ myeloid cells, together with a tendency towards increased numbers of pathogenic CD4^+^ T cells (**Figures 2E** **–G**). Among CNS-resident populations, we observed a reduction in O4^+^ immature oligodendrocytes and astrocytes (**Figure 2H** and **Supplementary Figures 2F, G** **)**. Together, these data demonstrate that PTN produced by astrocytes plays a major role in the resolution of late-stage CNS inflammation.

### Pleiotrophin Mitigates Pathogenic Glia Functions

**Figure 3:**
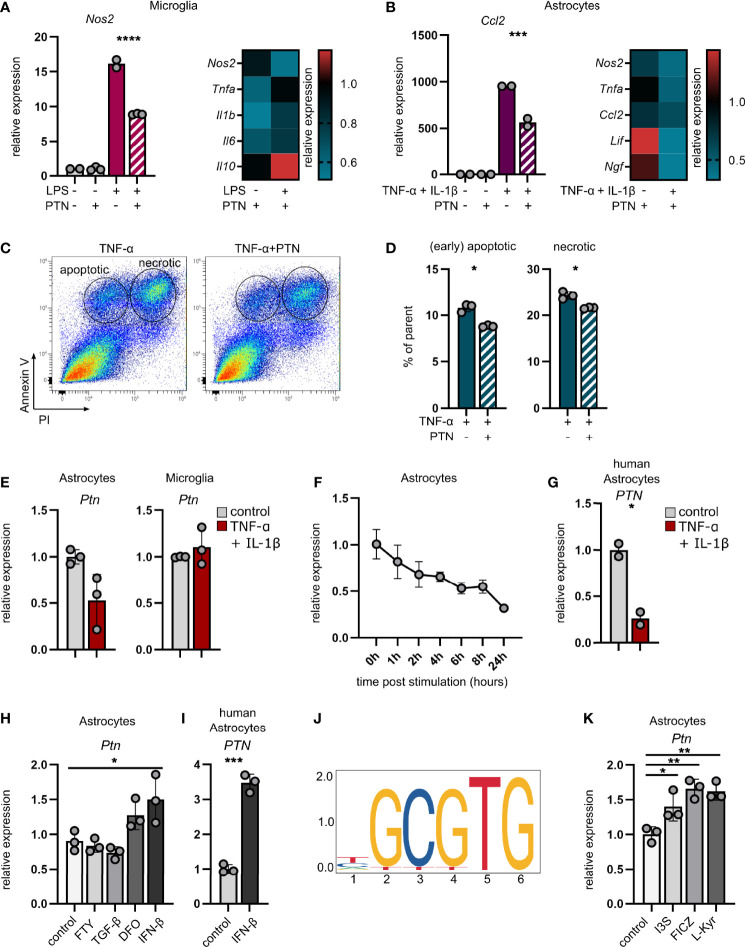
Pleiotrophin exerts anti-inflammatory properties and is regulated by AHR in astrocytes. **(A)** qPCR analysis of *Ptn* expression in unstimulated and LPS-stimulated microglia **(A)** and TNF-α/IL-1β-stimulated astrocytes **(B)** ± PTN. Heatmaps indicate the expression of indicated genes after PTN addition relative to their respective control without PTN. Data shown as mean ± SD. Unstim control *n* = 2, unstim + PTN *n* = 3, stim control *n* = 2, stim + PTN *n* = 3. Sidak’s multiple comparisons test. ***P < 0.001 ****P < 0.0001. **(C)** Representative scatterplots (entire group concatenated) and quantification **(D)** of apoptotic (Annexin V^+^PI^-^) and necrotic (Annexin V^+^PI^+^) N2A neuronal cells following stimulation with TNF-α ± PTN. Data (% of parent) shown as mean ± SD. stim control *n* = 3, stim + PTN *n* = 3. Unpaired t test with Welch’s correction. *P < 0.05. **(E)** qPCR analysis of *Ptn* expression in astrocytes (left) and microglia (right) sorted from CNS 24 hours after intracerebroventricular injection of TNF-α and IL-1β or vehicle (PBS). Data shown as mean ± SD. No stim (vehicle) *n* = 3, stim (TNF-α + IL-1β) *n* = 3. Unpaired t test with Welch’s correction. Astrocytes *P* = 0.0931. **(F)** qPCR analysis of *Ptn* expression in primary mouse astrocytes over the course of 24 hours following stimulation with TNF-α and IL-1β relative to unstimulated control. Data shown as mean ± SD. Unstim control *n* = 3, stim (per timepoint) *n* = 3. **(G)** qPCR analysis of *PTN* expression in primary human astrocytes following stimulation with TNF-α and IL-1β relative to an unstimulated control. Data shown as mean ± SD. Unstim control *n* = 2, stim *n* = 2. **(H)** qPCR analysis of *Ptn* expression in primary mouse astrocytes following stimulation with fingolimod (FTY), TGF-β, Deferoxamine (DFO) or IFN-β for 24 hours. Data shown as mean ± SD. *n* = 3 per stimulus. Sidak’s multiple comparisons test. *P < 0.05. **(I)** qPCR analysis of *PTN* expression in primary human astrocytes following stimulation with IFN-β for 24 hours. Data shown as mean ± SD. Unstim control *n* = 2, stim *n* = 2. Unpaired t test with Welch’s correction. *P < 0.05. **(J)** Putative AHR::ARNT binding site upstream of *Ptn*. **(K)** qPCR analysis of *Ptn* expression in primary mouse astrocytes following stimulation with the AHR agonists Indoxyl-3-sulfate (I3S), 6-formylindolo[3,2-b]carbazole (FICZ), L-Kynurenine for 24 hours. Data shown as mean ± SD. *n* = 3 per stimulus. Sidak’s multiple comparisons test. *P < 0.05, **P < 0.01, ***P < 0.001.

Based on our observations of increased inflammation in *Ptn* knockdown animals during EAE, we investigated whether PTN mediates anti-inflammatory effects on glial cells. Indeed, PTN reduced pro-inflammatory gene expression in activated primary mouse microglia and astrocytes (**Figures 3A, B** and **Supplementary Figures 3A, B** **)**. Furthermore, PTN increased neuronal survival following TNF-α challenge and reduced the number of apoptotic and necrotic neurons *in vitro* (**Figures 3C, D** and **Supplementary Figure 3C**).

In line with previously reported neurotrophic effects of PTN (22), these observations demonstrate that PTN mediates anti-inflammatory effects on astrocytes and microglia while enhancing neuronal survival under pro-inflammatory conditions. Consequently, enhancement of endogenous *Ptn* expression in astrocytes may be of relevance for the resolution of compartmentalized CNS inflammation.

In order to identify factors driving *Ptn* expression in inflammation-activated astrocytes, we simulated isolated CNS-intrinsic inflammation through intracerebroventricular injection of TNF-α and IL-1β, two cytokines known to effectively induce pro-inflammatory activation of astrocytes and microglia, and analyzed their *Ptn* expression. While astrocytes downregulated *Ptn* expression following cytokine injection, microglia showed no difference between cytokine and vehicle injected animals (**Figure 3E**), validating our observations that *Ptn* is regulated in astrocytes, but not in microglia upon CNS inflammation (**Figure 1B, C** **)**. This was recapitulated by *in vitro* stimulation of primary mouse and human astrocytes with TNF-α and IL-1β, demonstrating a downregulation of *Ptn* over the course of 24 hours (**Figure 3F, G** **)**, indicating that a pro-inflammatory environment per se does not induce the expression of *Ptn* in astrocytes.

Since TNF-α and IL-1β induce a pro-inflammatory activation state in astrocytes that is associated with pathogenic functions and the secretion of inflammatory cytokines, we next investigated whether potential counter-regulatory stimuli relevant for protective astrocyte polarization might induce the expression of *Ptn*, which we observed in our *in vivo* studies. Both interferon-β (IFN-β) and hypoxic conditions have previously been reported to induce the expression of *Ptn* in various cell types (23–25). Indeed, we observed upregulation of *Ptn* in primary mouse astrocytes under pseudohypoxic conditions induced by deferoxamine (DFO), as well as an induction of *Ptn* in mouse and human primary astrocytes by IFN-β, while neither transforming growth factor beta (TGF-β) nor the sphingosine-1-phosphate receptor modulator fingolimod (FTY) increased *Ptn* expression (**Figures 3H, I** **)**. In order to identify additional drivers of *Ptn* expression in astrocytes, we screened putative transcription binding factor sites in the *Ptn* promoter region using JASPER (26). Interestingly, we found numerous Ahr∷Arnt binding sites in a 60bp polyTG region of the mouse *Ptn* promoter, indicating that the ligand-activated aryl hydrocarbon receptor (AHR) may be a regulator of *Ptn* (**Figure 3J** and **Supplementary Figure 3E**). Indeed, stimulation of primary mouse astrocytes with the AHR agonists Indoxyl-3-sulfate (I3S), 6-Formylindolo(3,2-b)carbazole (FICZ) and L-Kynurenine (L-Kyn) induced the expression of *Ptn* (**Figure 3K**). Furthermore, we observed a reduction of *Ptn* expression in a previously published RNA-Seq dataset of AHR deficient astrocytes (GFAP-AhR) compared to controls (**Supplementary Figure 3F**) (27), collectively demonstrating that AHR activation induces *Ptn* expression. In total, these data demonstrate that astrocyte-derived *Ptn* expression is induced by factors that drive protective astrocyte activation states (27–29), supporting our observation of *PTN* upregulation in neuroprotective astrocyte subclusters in MS.

### Intranasal Delivery of PTN Attenuates Late-Stage EAE

Following our observations of anti-inflammatory and neuroprotective effects of PTN, we next sought to investigate the therapeutic potential of exogenously administered PTN in the context of late-stage neuroinflammation. Importantly, current therapeutic strategies in MS fail to recover late-stage inflammation and are predominantly efficient in acute relapsing-remitting MS.

**Figure 4:**
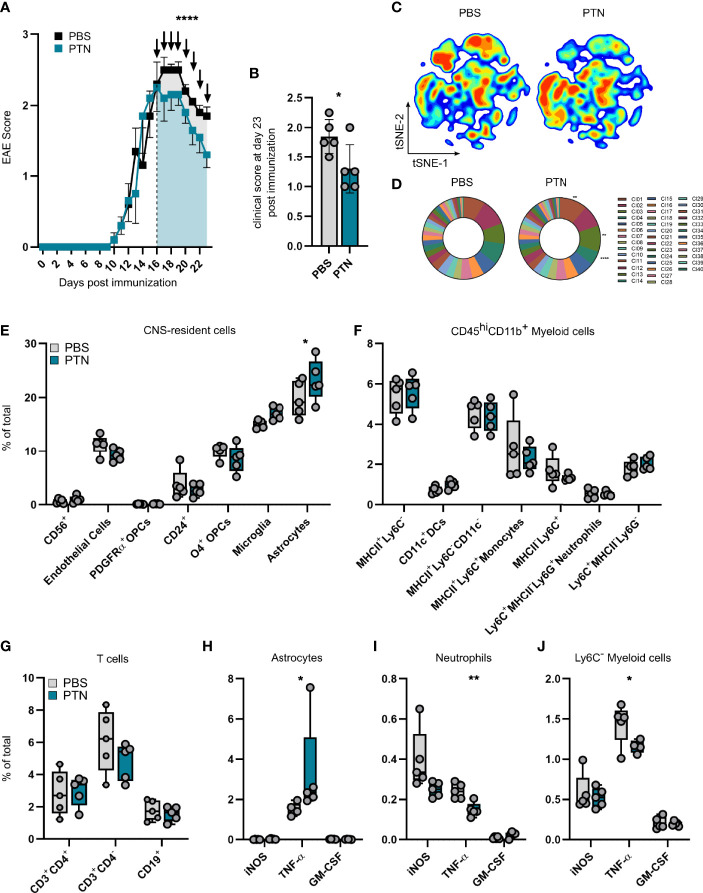
Intranasal treatment with PTN attenuates neuroinflammation and reduces pro-inflammatory signaling. **(A)** EAE progression in mice treated with vehicle (PBS, *n* = 5, black) or PTN (*n* = 5, blue) intranasally during late-stage EAE (starting day 17). Representative of two independent experiments. Data shown as mean ± S.E.M. Unpaired *t* test with Welch’s correction of area under the curve between day 16 and 23 (indicated by color). ****P < 0.0001. **(B)** Clinical score at day 23 post immunization. Data shown as mean ± SD. Vehicle-treated mice *n* = 5 (grey), PTN-treated mice *n* = 5 (blue). Unpaired *t* test with Welch’s correction. *P = 0.0608. **(C)** tSNE plot of CNS cells (brain and spinal cord) analyzed at day 23 post immunization by high-parameter flow cytometry. Vehicle-treated mice *n* = 5 (left), PTN-treated mice *n* = 5 (right). **(D)** Cluster abundance after PhenoGraph clustering of CNS cells (brain and spinal cord) analyzed at day 23 post immunization by high-parameter flow cytometry. Vehicle-treated mice *n* = 5 (left), PTN-treated mice *n* = 5 (right). Two-way repeated measures ANOVA. **P < 0.001, ****P < 0.0001. **(E)** Abundance of CNS-resident cell populations (brain and spinal cord), myeloid cells **(F)**, lymphocytes **(G)**, and their cytokine production **(H–J)** at day 23 post immunization analyzed by high-parameter flow cytometry. Data shown as mean ± SD. Vehicle-treated mice n = 5 (grey), PTN-treated mice *n* = 5 (blue). Sidak’s multiple comparisons test. *P < 0.05. **P < 0.01.

Thus, in order to study the relevance of PTN in late stages of CNS inflammation, we intranasally administered recombinant PTN in C57BL/6 mice starting at the peak of EAE, recapitulating a setting of clinical relevance in MS. Of note, intranasally applied therapeutics have been shown to effectively cross the blood brain barrier in rodents and primates and exert CNS-intrinsic effects by acting on glial cells (27, 30, 31). Seven days after treatment, mice receiving PTN showed decreased disease severity as compared to vehicle treated mice (**Figures 4A, B** **)**. Indeed, analysis by high-dimensional flow cytometry demonstrated significant differences in the cellular composition of CNS tissue derived from vehicle- or PTN-treated mice (**Figure 4C**). Dimensionality reduction and clustering using PhenoGraph revealed that major differences between groups were due to changes in CD45^-^CD11b^-^ CNS cells (**Figure 4D** and **Supplementary Figures 4A, B** **)**. Indeed, among CNS-resident cell populations, the number of astrocytes and microglia was increased, while no differences in abundance within the myeloid or lymphocyte compartment were detected (**Figures 4E** **–G**). Interestingly, astrocytes and microglia produced enhanced levels of TNF-α, while myeloid cells reduced their TNF-α production (**Figures 4H–J** and **Supplementary Figures 4E, F** **)**. Of note, CNS targeting application of PTN did not alter peripheral immune cell composition (**Supplementary Figure 5**). Together, intranasal application of PTN in previously established CNS inflammation mitigates late stages of EAE due to its effects on glial cells and their production of neurotoxic mediators.

## Discussion

Here, we describe astrocytes as major producers of PTN in the context of neuroinflammation. We demonstrate that astrocyte-derived PTN promotes recovery in late-stage autoimmune CNS inflammation and exerts previously unknown anti-inflammatory effects on glial cells. Furthermore, we show that intranasally administered PTN attenuates late-stage EAE and may serve as potential novel treatment strategy for late stages of MS.

Although several cell types have previously been described as cellular sources of PTN, the role of astrocytes as PTN producers and particularly its function in the context of neuroinflammation has only vaguely been illuminated (16, 22, 32). Early evidence for a role of PTN in the context of autoimmune CNS inflammation comes from a study by Liu et al. (33), in which the authors observe an increase of PTN levels in spinal cord tissue of Lewis rats during late-stage EAE, but did not pinpoint a producer or target cell. Since then, numerous studies have suggested angiogenic and anti-inflammatory effects of PTN in the peripheral immune compartment (10, 34–36). In malignant CNS disorders including glioblastoma, PTN secreted by neural precursors as well as tumor-associated macrophages promotes glioma growth and invasion into the subventricular zone (37, 38), suggesting a role of PTN in cell proliferation and survival of CNS resident cells. However, the role of PTN in inflammatory CNS disorders and their late stages remains unclear.

We here describe upregulation of *PTN* expression and PTN immunoreactivity in human and mouse astrocytes during acute and late-stage CNS inflammation, matching early findings in the context of neuronal injury and ischemia (16, 25). *Ptn* upregulation following peripherally-induced CNS inflammation was localized to upper and lower cortical layers, as demonstrated in a recent study by Hasel et al. (4). This is particularly interesting as cortical inflammation and grey matter damage have been described to closely correlate with disease progression and physical disability in MS (20, 39–41). In this context, our findings of AHR regulation of *Ptn* expression might be of high relevance, as data from the BRAVO Phase III trial demonstrate the highest reduction in grey matter (GM) volume decrease following treatment with the AHR-agonist Laquinimod (Laq), compared to IFN-β- and Placebo-treated patients (42). This was further recapitulated in the recent CONCERTO trial, in which oral Laq treatment led to a significant reduction in brain volume loss compared to the placebo group, as well as a numerical reduction in time to first relapse and annualized relapse rate as exploratory endpoints, while it did not alter 3-month confirmed disability progression (43). Together with observations of protective astrocyte signaling following oral administration of Laq during EAE (28), one may speculate that this positive effect of Laq on GM loss may, at least to some extent, be carried by increased levels of PTN, which limits inflammatory signaling in glial cells and supports neuronal survival, as we have demonstrated *in vitro* and *in vivo*. However, a detailed examination of brain tissue obtained from Laq-treated MS patients would be needed to confirm this hypothesis. Our finding that AHR is a transcriptional regulator of astrocytic *Ptn* expression is further supported by reduced serum levels of AHR-agonists in MS patients during remission and primary progressive MS (PPMS), matching our observations of diminished astrocyte-derived PTN expression during late-stage EAE (44, 45). In this line, we speculate that reduced AHR signalling in chronic stages of MS leads to the loss of neuroprotective astrocyte activation states, accompanied by a reduction in astrocyte-derived PTN in cortical layers, which ultimately results in the perpetuation of CNS inflammation and disease progression.

Intriguingly, both our CRISPR-Cas9-mediated knockdown model and treatment approach demonstrate a strong effect of PTN on the production of TNF-α by CNS-resident and infiltrating cells. While a reduction in astrocyte-derived PTN increased the production of TNF-α by monocytes and Ly6C^-^ myeloid cells, exogenous administration of PTN successfully reduced the amount of TNF-α produced by myeloid cells. Conversely, astrocytes and microglia exhibited a tendency towards decreased TNF-α in Gfap-*Ptn* mice, while intranasal administration of PTN increased TNF-α produced by astrocytes and microglia. These opposing effects exemplify the multifaceted roles of TNF-α signaling in CNS and periphery, as has been described previously (46, 47; J. 48–51). Indeed, early studies demonstrate that TNF receptor (TNFR)-signaling in the acute phase of EAE drives inflammation, while at later stages it exerts protective functions (52; J. 48). These effects have also been observed in several other models of neurodegeneration and -inflammation and can be explained through the divergent roles of TNFR1 and TNFR2 (46, 53–55). Future experiments will be needed to examine the receptors and downstream signals that modulate the PTN-driven regulation of TNF signaling and its differential roles on CNS-resident and infiltrating cells.

In summary, we have identified astrocytes as novel source of PTN in the context of neuroinflammation and demonstrated its relevance for the resolution of CNS inflammation in a preclinical model of MS. Our findings demonstrate a previously unknown regulation of PTN by AHR and provide first evidence of a therapeutic role of PTN in the resolution of late-stage neuroinflammation. Altogether, these data may drive the development and understanding of novel therapeutic strategies for the treatment of chronic progressive MS.

## Material and Methods

### Mice

Mice were housed two to five animals per cage under a standard light cycle (12 h:12 h light:dark) (lights on from 07:00 to 19:00) at 20–23 °C and humidity (~50%) with ad libitum access to water and food. Adult female mice 8–12 weeks old and P0–P3 pups were used on a C57Bl/6J background (The Jackson Laboratory, #000664).

### EAE

EAE was induced in 8-12 week female C57Bl/6J mice using 150 μg of MOG_35–55_ (Genemed Synthesis, 110582) mixed with freshly prepared complete Freund’s Adjuvant [using 20 ml Incomplete Freund’s Adjuvant (BD Biosciences, #BD263910) mixed with 100 mg Myobacterium tuberculosis H37Ra (BD Biosciences, #231141)] at a ratio of 1:1 (v/v at a concentration of 5 mg ml−1). All mice received 2 subcutaneous injections of 100 μl each of the MOG_35-55_/CFA mix. All mice then received a single intraperitoneal injection of 200 ng pertussis toxin (List Biological Laboratories, #180) at a concentration in 200 μl of PBS. Mice received a second pertussis toxin injection at the same concentration two days after EAE induction. Mice were monitored and scored daily thereafter. EAE clinical scores were defined as follows: 0, no signs; 1, fully limp tail; 2, hindlimb weakness; 3, hindlimb paralysis; 4, forelimb paralysis; 5, moribund.

Intranasal delivery of PTN or vehicle (PBS) was started on day 16 after EAE induction. 20 µl of vehicle or 300µg/kg PTN (solved in PBS) was applied drop by drop on nostrils.

### Lentivirus Production

Lentiviral vectors were produced as previously described (6, 7, 56). Lentiviral vectors were obtained from lentiCRISPRv2 (Addgene #5296155), and lentiCas9-EGFP (Addgene #6359256). sgRNAs were substituted through a three-way cloning strategy using the following primers: U6-PCR-F 5′-AAAGGCGCGCCGAGGGCCTATTT-3′, U6-PCR-R 5′-TTTTTTGGTCTCCCGGTGTTTCGTCCTTTCCAC-3′, cr-RNA-F 5′-AAAAAAGGTCTCTACCG(sgRNA)GTTTTAGAGCTAGAAATAGCAAGTT-3′, cr-RNA-R 5′-GTTCCCTGCAGGAAAAAAGCACCGA-3. Products were amplified using Phusion Master Mix (Thermo Fisher Scientific Scientific, #F548S) and purified using the QIAquick PCR Purification Kit (Qiagen, 28104), followed by digestions using DpnI (NEB, #R0176S), BsaI-HF (NEB, #R3535/R3733), AscI (NEB, #R0558), or SbfI-HF (NEB, #R3642). Ligations were performed overnight at 16 °C using T4 DNA Ligase Kit (NEB, #M0202L). Ligations were transformed into NEB Stable Cells (NEB, #C3040) at 37 °C and single colonies were picked the following day. Plasmid DNA was isolated using QIAprep Spin Miniprep Kit (Qiagen, #27104) and the lentiviral plasmids were transfected into HEK293FT cells according to the ViraPower Lentiviral Packaging Mix protocol (Thermo Fisher Scientific Scientific, #K497500) with pLP1, pLP2, and pseudotyped with pLP/VSVG. Medium was changed the next day, lentivirus was collected 48 h later and concentrated using Lenti-X Concentrator (Clontech, #631231) according to the manufacturer’s protocol. Concentrates were resuspended in 1/100 of the original volume in PBS. Delivery of lentiviruses *via* intracerebroventricular (i.c.v.) injection was performed as described previously (6, 7, 56). In brief, mice were anaesthetized using 1% isoflurane mixed with oxygen. Heads were shaved and cleaned using 70% ethanol and Lidocain-gel followed by a medial incision of the skin to expose the skull. The ventricles were targeted bilaterally using the coordinates: ± 1.0 (lateral), −0.44 (posterior), −2.2 (ventral) relative to Bregma. Mice were injected with approximately 10^7^ total IU of lentivirus delivered by two 10 μl injections using a 10 μl Hamilton syringe (Sigma-Aldrich, #20787) on a Stereotaxic Alignment System (Kopf, #1900), sutured, and permitted to recover in a separate clean cage. Mice received a subcutaneous injection of 1mg/kg Meloxicam post i.c.v. injection and 48 h later. Mice were permitted to recover for between 4 and 7 days before induction of EAE. CRISPR–Cas9 sgRNA sequences were designed using a combination of the Broad Institute’s sgRNA GPP Web Portal (https://portals.broadinstitute.org/gpp/public/analysis-tools/sgrna-design), Synthego (https://design.synthego.com/#/validate). sgRNAs used in this study were: *Ptn*: 5′-AGTGTGTGCGTGCCTACCAG-3′; *Scrmbl* 5-GCACTACCAGAGCTAACTCA-3′.

### Cell Culture Experiments and Stimulants

If not stated otherwise, the following concentrations were used for stimulation experiments: mouse PTN (R&D, #6580-PL-050) 50 ng/ml, mouse TNF-α (Peprotech, #AF-315-01A) 50 ng/ml, mouse IL-1β (Peprotech, #211-11B), mouse IFN-β (R&D, #8234-MB-010/CF) 1.000 U/ml, mouse TGF-β (R&D, #7666-MB-005/CF) 10ng/ml, FTY720 (Sigma, #SML0700-5MG) 10 µM, DFO (Merck, #252750-1gm) 200µM, I3S (Sigma, #I3875-250MG) 50 µg/ml, FICZ (Sigma, #SML1489-1MG) 1µM, L-Kynurenin (Sigma, #K8625-25MG) 1µM, human TNF-α (Peprotech, #300-01A) 50 ng/ml, human IL-1β (Peprotech, #200-01B) 100 ng/ml, human IFN-β (Peprotech, #300-02BC) 1.000 U/ml.

### Primary Mouse Astrocyte and Microglia Cultures and Stimulation Experiments

Brains of mice aged P0–P3 were dissected into PBS on ice. Brains of 6-8 mice were pooled, centrifuged at 500g for 10 min at 4 °C and resuspended in 0.25% Trypsin-EDTA (Thermo Fisher Scientific Scientific, #25200-072) at 37 °C for 10 min. DNase I (Thermo Fisher Scientific Scientific, #90083) was added at 1 mg/ml to the solution, and the brains were digested for 10 more minutes at 37 °C. Trypsin was neutralized by adding DMEM + GlutaMAX (Thermo Fisher Scientific Scientific, #61965026) supplemented with 10% FBS (Thermo Fisher Scientific Scientific, #10438026) and 1% penicillin/streptomycin (Thermo Fisher Scientific Scientific, #10500064), and cells were passed through a 70-µm cell strainer. Cells were centrifuged at 500g for 10 min at 4 C, resuspended in DMEM + GlutaMAX with 10% FBS 1% penicillin/streptomycin and cultured in T-75 flasks (Sarstedt, #83.3911.002), pre-coated with 2 µg/ml Poly-L Lysine (PLL, Provitro, #0413) at 37 °C in a humidified incubator with 5% CO2 for 5–7 days until confluency was reached. Mixed glial cells were shaken for 30 min at 180 rpm, the supernatant was collected and the medium was changed, and then cells were shaken for at least 2 h at 220 rpm and the supernatant was collected and the medium was changed again. CD11b^+^ microglia were isolated from the collected supernatant using the CD11b Microbead Isolation kit (Miltenyi, #130-049-601) according to the manufacturer’s instruction. For stimulation experiments, astrocytes and microglia were detached using TrypLE (Thermo Fisher Scientific, #12604013) and seeded in PLL-coated 48-well plates (Sarstedt, #NC1787625) at a density of 150.000 cells per well.

### Primary Human Astrocyte Cultures and Stimulation Experiments

Primary human astrocytes were obtained from ScienCell (#1800) and cultured according to the manufacturer’s instructions. In brief, cells were passaged in astrocyte medium (ScienCell, #1801) until confluency and subsequently plated onto plates pre-coated with 2 µg/ml Poly-L Lysine (Provitro, #0413). For stimulation experiments, astrocytes were detached using TrypLE (Thermo Fisher Scientific, #12604013) and seeded in PLL-coated 48-well plates (Sarstedt, #NC1787625) at a density of 150000 cells per well.

### Neuronal Cultures and Stimulation Experiments

N2A Neuronal cells (ATCC CCL-131, ATCC, Manassas) were seeded in 24 well-plates (Sarstedt, #NC0984607) at a density of 200.000 cells per well and activated with 20ng/ml mTNF-α for 24h with or without the addition of PTN. Following 24h stimulation, cells were harvested using TrypLE (Thermo Fisher Scientific, #12604013) and apoptosis was assessed using the Annexin V APC Kit (Biolegend, #640919) according to the manufacturer’s instruction.

### T Cell Differentiation and Stimulation Experiments

T cells were isolated from spleen and lymph nodes of naïve C57BL6/J mice as previously described (6). Naïve CD4^+^ T cells were purified using the Naive CD4+ T Cell Isolation Kit (Miltenyi, #130-104-453) according to the manufacturer’s instruction. Naive cells were cultured at a concentration of 1.0–1.5 × 106/ml. Cells were stimulated in the presence of plate bound anti-CD3 Ab (1–3 μg/ml, BioXCell #BE0002) and anti-CD28 Ab (1–2 μg/ml, BioXCell #BE0015-5). For the generation of T_H_17 cells, naive T cells were cultured with IL-6 (30 ng/ml, R&D #406-ML-005), TGF-β (3 ng/ml), anti-IFNγ (10 µg/ml, BioXCell #BE0055-1MG), and anti-IL-4 (10 µg/ml, BioXCell #BE0045-1MG). After 48h, T_H_17 cells were supplemented with 10 ng/ml IL-23. For T_H_1 cells, naïve CD4^+^ T cells were culture in the presence of IL-12 (10 ng/ml, R&D #419-ML-010) and anti-IL-4 (10 µg/ml). For T_Reg_, cells were cultured in the presence of TGF-β (5 ng/ml), anti-IFNγ (10 µg/ml), and anti-IL-4 (10 µg/ml). After 3 days, recombinant mouse PTN or vehicle was added. After 4 days, cytokine was measured by intracellular cytokine staining (using the Foxp3/Transcription Factor Staining Buffer Set, eBiosciences #00-5523-00) and subsequent flow cytometry.

### Isolation of Cells From Adult Mouse CNS

Mice were perfused with cold 1× PBS and the CNS was isolated and mechanically diced using sterile razors. Brains and spinal cords were pooled and transferred into 5 ml of enzyme digestion solution consisting of 35.5 µl papain suspension (Worthington, #LS003126) diluted in enzyme stock solution (ESS) and equilibrated to 37 °C. ESS consisted of 10 ml 10× EBSS (Sigma-Aldrich, #E7510), 2.4 ml 30% D(+)-glucose (Sigma-Aldrich, #G8769), 5.2 ml 1 M NaHCO3 (VWR, #AAJ62495-AP), 200 µl 500 mM EDTA (Thermo Fisher Scientific Scientific, #15575020), and 168.2 ml ddH2O, filter-sterilized through a 0.22-µm filter. Samples were shaken at 80 rpm for 30–40 min at 37 °C. Enzymatic digestion was stopped with 1 ml of 10× hi ovomucoid inhibitor solution and 20 µl 0.4% DNase (Worthington, #LS002007) diluted in 10 ml inhibitor stock solution (ISS). 10× hi ovomucoid inhibitor stock solution contained 300 mg BSA (Sigma-Aldrich, #A8806) and 300 mg ovomucoid trypsin inhibitor (Worthington, #LS003086) diluted in 10 ml 1× PBS and filter sterilized using a 0.22-µm filter. ISS contained 50 ml 10× EBSS (Sigma-Aldrich, #E7510), 6 ml 30% D(+)-glucose (Sigma-Aldrich, #G8769), and 13 ml 1 M NaHCO3 (VWR, #AAJ62495-AP) diluted in 170.4 ml ddH2O and filter-sterilized through a 0.22-µm filter. Tissue was mechanically dissociated using a 5-ml serological pipette and filtered through a 70-µm cell strainer (Fisher Scientific, #22363548) into a fresh 50-ml conical tube. Tissue was centrifuged at 600g for 5 min and resuspended in 10 ml of 30% Percoll solution (9 ml Percoll (GE Healthcare Biosciences, #17-5445-01), 3 ml 10× PBS, 18 ml ddH2O). Percoll suspension was centrifuged at 600g for 25 min with no breaks. Supernatant was discarded and the cell pellet was washed once with 1× PBS, centrifuged at 500g for 5 min and prepared for downstream applications.

### Flow Cytometry

Live/dead staining was performed with LIVE/DEAD™ Fixable Aqua Dead Cell Stain Kit (Thermo Fisher Scientific Scientific, #L34957) according to the manufacturer’s instructions. Cells were subsequently stained at 4°C in the dark for 20 min with flow cytometry antibodies, diluted in FACS buffer (1x PBS, 2% FBS, 2mM EDTA). Cells were then washed twice with FACS buffer and resuspended in 1× PBS for acquisition. Antibodies used in this study were: BV421-CD11b (Biolegend, #101235), eF450-CD3 (Thermo Fisher Scientific Scientific, # 48-0031-80), BV480-CD11c (BD, #565627), BV510-F4/80 (Biolegend,# 123135), BV570-Ly6C (Biolegend,# 128029), BV605-CD80 (BD, #563052), BV650-CD56 (BD, #748098), BV650-CD8 (Biolegend, #100741), PE-eFlour610-CD140a (Thermo Fisher Scientific Scientific, #61140180), SuperBright780-MHCII (Thermo Fisher Scientific Scientific, #78532080), BV711-CD74 (BD, #740748), PE-B220 (BD, #561878), PE-CD105 (Thermo Fisher Scientific Scientific, # 12-1051-82), AF488-A2B5 (Novus Biologicals, #FAB1416G), PE-Cy5-CD24 (Biolegend, #101811), PerCP-eFlour710-CD86 (Thermo Fisher Scientific Scientific, #46086280), AF532-CD44 (Thermo Fisher Scientific Scientific, #58044182), PE-Cy5.5-CD45 (Thermo Fisher Scientific Scientific, #35045180), JF646-MBP (Novus Biologicals, #NBP2-22121JF646), APC-Cy7-Ly6G (Biolegend, #127623), AF700-O4 (R&D, #FAB1326N), BUV737-CD154 (BD, #741735), AF660-CD19 (Thermo Fisher Scientific Scientific, #606019380), APC/Fire810-CD4 (Biolegend, #100479).

For intracellular flow cytometry staining, cells were fixed over night after surface staining using the eBioscience™ Foxp3/Transcription Factor Staining Buffer Set (eBioscience, #00552300) according to the manufacturer’s instructions. For staining of intracellular cytokines, the following antibodies were used: PE-eFlour610-iNOS (eBioscience, #61592080), BV711-IL17a (Biolegend, #506941), PE-CCl2 (BD, #554443), PE-Cy5-FoxP3 (Thermo Fisher Scientific Scientific, #15-5773-82), PE-Cy7-IFNγ (Biolegend, #505826), PE PerCP-eFlour710-TNF (eBioscience, #46732180), APC-GM-CSF (eBioscience, #17733182), APC-eF780-Ki67 (Thermo Fisher Scientific Scientific, #506941).

### Analysis of Multiparameter Flow Cytometry Data

Data was analyzed using the OMIQ platform. In brief, cells were gated as described in **Supplementary Figure 6** and subsampled to a set number of cells per group. Opt-SNE (max. 1000 iterations, perplexity 30, theta 0.5, verbosity 25) or UMAP (15 neighbors, minimum distance 0.4, 200 Epochs) was performed, followed by PhenoGraph clustering (based on Euclidian distance). SAM (21) was performed on groups using a two-class unpaired approach when two groups were compared (with max. 100 permutations and a FDR cutoff of 0.1).

### RNA Isolation and RT-qPCR

Cells were lysed in 350µl RLT buffer (Qiagen) and RNA was isolated using the RNeasy Mini Kit (Qiagen, #74004) according to the manufacturer’s instructions. 500 ng RNA of each sample was transcribed into cDNA using the High-Capacity cDNA Reverse Transcription Kit (Life Technologies, #4368813). Gene expression was assessed by qPCR using the TaqMan Fast Advanced Master Mix (Life Technologies, #4444556). The following TaqMan-probes were used: *Actb* (Mm02619580_g1), *Bdnf* (Mm04230607_s1), *Ccl2* (Mm00441242_m1), *Gapdh* (Mm99999915_g1), *Il6* (Mm00446190_m1), *Il10* (Mm01288386_m1), *Lif* (Mm00434762_g1), *Ngf* (Mm00443039_m1), *Nos2* (Mm00440502_m1), *Ptn* (Mm01132688_m1), *Tnf* (Mm00443258_m1), *PTN* (Hs01085691_m1). qPCR data were analyzed by the ΔΔCt method.

### Immunohistochemistry

For immunohistochemical analyses, mice were transcardially perfused with cold PBS. After perfusion, lumbar spinal cord (L1-L6) was dissected and processed for immunofluorescence labelling. The tissue was post-fixed in 4% PFA/1x PBS for 24 hours. After post-fixation, the spinal cords were dehydrated at 4° C in 30% sucrose in PBS overnight. By means of liquid nitrogen-cooled 2-methylbutane, the tissue was frozen in tissue-Tek embedding medium and kept at -20° C for storage. For the visualization and quantitative studies of PTN expressing astrocytes and microglia over the course of EAE, 10μm thick cross cryostat serial sections (Leica) of spinal cords were obtained on glass slides and stored at -20°C. Spinal cords were then stained for PTN, GFAP and IBA-1. Cross sections were incubated in 4% PFA for 10 minutes at room temperature for post-fixation. After washing the slides in 1x PBS for five minutes, they were incubated in blocking buffer (5% BSA/10% donkey serum/0.3% Triton-X/1x PBS) for 45 minutes. Slides were incubated overnight at 4°C with rat anti-GFAP (1:1000; Life Technologies; # 13-0300), rabbit anti-IBA1 (1:800; Abcam; #ab178846), mouse anti-PTN (1:100; Santa Cruz; #sc-74443) diluted in 1% BSA/1% donkey serum/0.3% Triton-X/1x PBS. On the following day, three washing steps of five minutes each preceded the incubation with the secondary antibodies for one hour: goat anti-rat IgG Cy3 (1:500; Thermo Fischer; A10522), donkey anti-rabbit IgG AF647 (1:500; Dianova; #711-605-152) and donkey anti-mouse IgG AF488 (1:500; Life Technologies; #A21202). During the further procedure, sections were washed three times for five minutes before and after the 10-minute incubation with DAPI (1:100.000). After this process, the slides were cover-slipped with Prolong Gold anti-fade and stored at 4°C for further analysis.

### Evaluation of Immunohistochemical Results

Images of immunofluorescent labelled sections were acquired using the software Zen 3.0 (blue edition). For quantification, staining against PTN, GFAP and IBA1 was examined in three distinct regions of the spinal cord (posterior column, anterolateral column, grey matter) using a fluorescence microscope (Axio Observer Z1, Zeiss) at 20x magnification. GFAP+ and IBA1+cells were quantified manually in an unbiased manner by the same investigator. The number of quantified cells was related to the area of spinal cord using the cell counter plugin in ImageJ software (Rasband, W.S, ImageJ, National Institutes of Health, Bethesda, Maryland, USA). The areas of the evaluated spinal cord preparations were measured with the ImageJ software. Image processing was performed using Photoshop CS6 (Adobe).

### Prediction of Transcription Factor Binding Sites

Putative transcription factor binding sites were identified using JASPER (26) (https://jaspar.genereg.net/) on the 1.000 bp upstream region of the mouse *Ptn* locus with a 80% relative profile score threshold.

### Pathway Analysis

GSEAPreranked analyses were used to generate enrichment plots for scRNA-seq data using MSigDB molecular signatures for canonical pathways: KEGG/Reactome/Biocarta (c2.cp.all).

### Single-Cell RNA Seq Analysis

Data including statistics of human astrocytes was obtained from Wheeler et al. (7) and visualized using matplotlib.pyplot. tSNE plots and Visium spatial transcriptome plots were obtained from https://liddelowlab.shinyapps.io/GliaSeqPro/ with original data from Hasel et al. (4).

## Data Availability Statement

The public scRNA-Seq datasets are published by Wheeler et al. (7) and Hasel et al. (4) and can be found under the Gene Expression Omnibus (GEO) SuperSeries accession number GSE130119 and GSE148612.

## Ethics Statement

The animal study was reviewed and approved by Bavarian State Authorities (Regierung von Unterfranken, AZ 55.2.2-2532-2-1306).

## Author Contributions

ML performed most *in vitro*, and *in vivo* experiments. LL, DF, OV, TT, and UN assisted with *in vitro* and *in vivo* experiments. LL, and UN performed immunohistochemical analyses. ML, DF, and VR wrote the manuscript with input from coauthors. ML and VR designed the study and edited the manuscript. All authors contributed to the article and approved the submitted version.

## Funding

ML and VR were funded by an ERC Starting Grant by the European research Council (HICI 851693). VR was supported by a Heisenberg fellowship and Sachmittel support provided by the German Research Foundation (Deutsche Forschungsgemeinschaft, DFG, RO4866-3/1, RO4866-4/1) as well as in transregional and collaborative research centers provided by the German Research Foundation (DFG, Project ID 408885537 - TRR 274; Project ID 261193037 - CRC 1181). TT was funded by the Kommission für Klinische Forschung (KKF), Klinikum rechts der Isar. LL was funded by transregional research center provided by the German Research Foundation (DFG, German Research Foundation - Project ID 408885537 - TRR 274). The authors report no targeted funding.

## Conflict of Interest

The authors declare that the research was conducted in the absence of any commercial or financial relationships that could be construed as a potential conflict of interest.

## Publisher’s Note

All claims expressed in this article are solely those of the authors and do not necessarily represent those of their affiliated organizations, or those of the publisher, the editors and the reviewers. Any product that may be evaluated in this article, or claim that may be made by its manufacturer, is not guaranteed or endorsed by the publisher.

## Supplementary Material

Supplementary Figure 1
**(A)** Abundance of astrocyte clusters as determined in Wheeler et al. (7) in control and MS patients. **(B)** Gene-set enrichment analysis of cluster 4 (KEGG hsa04722). **(C)** tSNE plot and Visium spatial expression **(D)** of *Ptn* expression in astrocyte subclusters following peripheral LPS- or vehicle (CONT)-injection obtained from Hasel et al. (4).

Supplementary Figure 2
**(A)** Immunostaining and quantification **(B)** of GFAP^+^PTN^+^ astrocytes in lumbar spinal cord pf Gfap-Scrmbl and Gfap-Ptn mice at day 28 post immunization. Scale bar represents 100 µm. Data shown as mean (*n* = 3) ± SD. Unpaired t test with Welch’s correction. * *P* < 0.05. **(C)** Median fluorescent intensities (MFIs) of activation markers expressed on CD45^hi^CD11b^+^ myeloid cells in CNS (brain and spinal cord) of Gfap-Scrmbl (*n* = 4) and Gfap-Ptn (n = 5) animals quantified by flow cytometry. **(D, E)** Significance Analysis of Microarrays (SAM) of cell populations in Gfap-Scrmbl (*n* = 4) and Gfap-Ptn (*n* = 5) mice at day 28 post immunization. **(F)** Cytokine production by astrocytes and microglia **(G)** in Gfap-Scrmbl (*n* = 4) and Gfap-Ptn (*n* = 5) mice at day 28 post immunization quantified by intracellular flow cytometry. Data shown as mean ± SD.

Supplementary Figure 3
**(A)** qPCR analysis of *Ptn* expression in unstimulated and LPS-stimulated microglia **(A)** and TNF-α/IL-1β-stimulated astrocytes **(B)** ± PTN. Data shown as mean ± SD. Unstim control *n* = 2, unstim + PTN *n* = 3, stim control *n* = 2, stim + PTN *n* = 3. Sidak’s multiple comparisons test. *** *P* < 0.001. **(C)** Quantification of live (Annexin V^-^PI^-^) N2A neuronal cells following stimulation with TNF-α ± PTN. Data shown as mean ± SD. Stim control *n* = 3, stim + PTN = 3. Unpaired *t* test with Welch’s correction. * *P* < 0.05. **(D)** Quantification of T cell subsets following differentiation ± PTN. Data shown as mean ± SD. Control *n* = 2, stim + PTN = 2. Unpaired *t* test with Welch’s correction. **(E)** Ahr : Arnt binding sites in the promoter regions upstream of the *Ptn* coding region, analyzed with JASPER (57). **(F)** FPKM values of *Ptn* expression in sorted astrocytes of control and Gfap-Ahr animals as obtained from Rothhammer et al. (27).

Supplementary Figure 4
**(A)** tSNE plot of CNS cells (brain and spinal cord) obtained from vehicle-treated (*n* = 5) or PTN-treated (*n* = 5) animals, quantified by high-parameter flow cytometry. Clusters were determined using PhenoGraph. **(B)** Marker expression (MFI) in respective clusters in pooled vehicle-treated and PTN-treated animals. **(C)** tSNE plot of CNS cells (brain and spinal cord) obtained from vehicle-treated (*n* = 5) or PTN-treated (*n* = 5) animals, quantified by high-parameter flow cytometry. Clusters were determined using manual gating. **(D)** Abundance of CD3^+^CD4^+^ T cell subsets in CNS (brain and spinal cord) of vehicle-treated (*n* = 5, grey), and PTN-treated (*n* = 5, blue) mice. **(E)** Cytokine production microglia and CD45^hi^CD11b^+^Ly6C^+^ monocytes **(F)** in CNS (brain and spinal cord) of vehicle-treated (*n* = 5, grey), and PTN-treated (*n* = 5, blue) mice. Sidak’s multiple comparisons test. *** *P* < 0.001.

Supplementary Figure 5
**(A)** UMAP plot of splenic cells from vehicle-treated (n = 5) and PTN-treated (*n* = 5) mice at day 23 post immunization quantified by high-parameter flow cytometry. **(B)** Abundance of splenic cells from vehicle-treated (*n* = 5, grey) and PTN-treated (*n* = 5, blue) mice at day 23 post immunization quantified by high-parameter flow cytometry. **(C)** Median fluorescent intensities (MFIs) of activation markers expressed by CD11c^+^ dendritic cells, Ly6G^-^ monocytes, and Ly6G^+^ neutrophils in spleens of vehicle-treated (*n* = 5, dark grey) and PTN-treated (n = 5, light grey) mice at day 23 post immunization. **(D)** Abundance of T cell subsets in spleens from vehicle-treated (*n* = 5, grey) and PTN-treated (*n* = 5, blue) mice at day 23 post immunization quantified by flow cytometry. **(E)** Quantification of cytokine production by Ly6G^-^ monocytes, Ly6G^+^ Neutrophils **(F)**, and CD45^hi^CD11b^+^Ly6C^-^ myeloid cells **(G)** in spleens of vehicle-treated (*n* = 5, grey) and PTN-treated (*n* = 5, blue) mice at day 23 post immunization.

Supplementary Figure 6
**(A)** Gating strategy used for high-parameter flow cytometry surface staining of CNS cells. Astrocytes were identified by a negative gating strategy as described previously (27, 58, 59). **(B)** Gating strategy used for high-parameter flow cytometry surface staining of splenic cells. **(C)** Gating strategy used for intracellular staining of CNS cells. Following live/dead discrimination, a dump channel was used to exclude erythrocytes (TERR119), B cells (B220), OPCs (CD140a), Neutrophils (Ly6G). Astrocytes were identified by negative gating. For splenocyte intracellular staining, the same strategy was applied with the inclusion of CD19/B220.
