# Supplementary figures and images for: Astrocyte-Derived Pleiotrophin Mitigates Late-Stage Autoimmune CNS Inflammation
Source: Front Immunol. 2022 Jan 3;12:800128. doi: 10.3389/fimmu.2021.800128 (PMC8762329; doi:10.3389/fimmu.2021.800128)

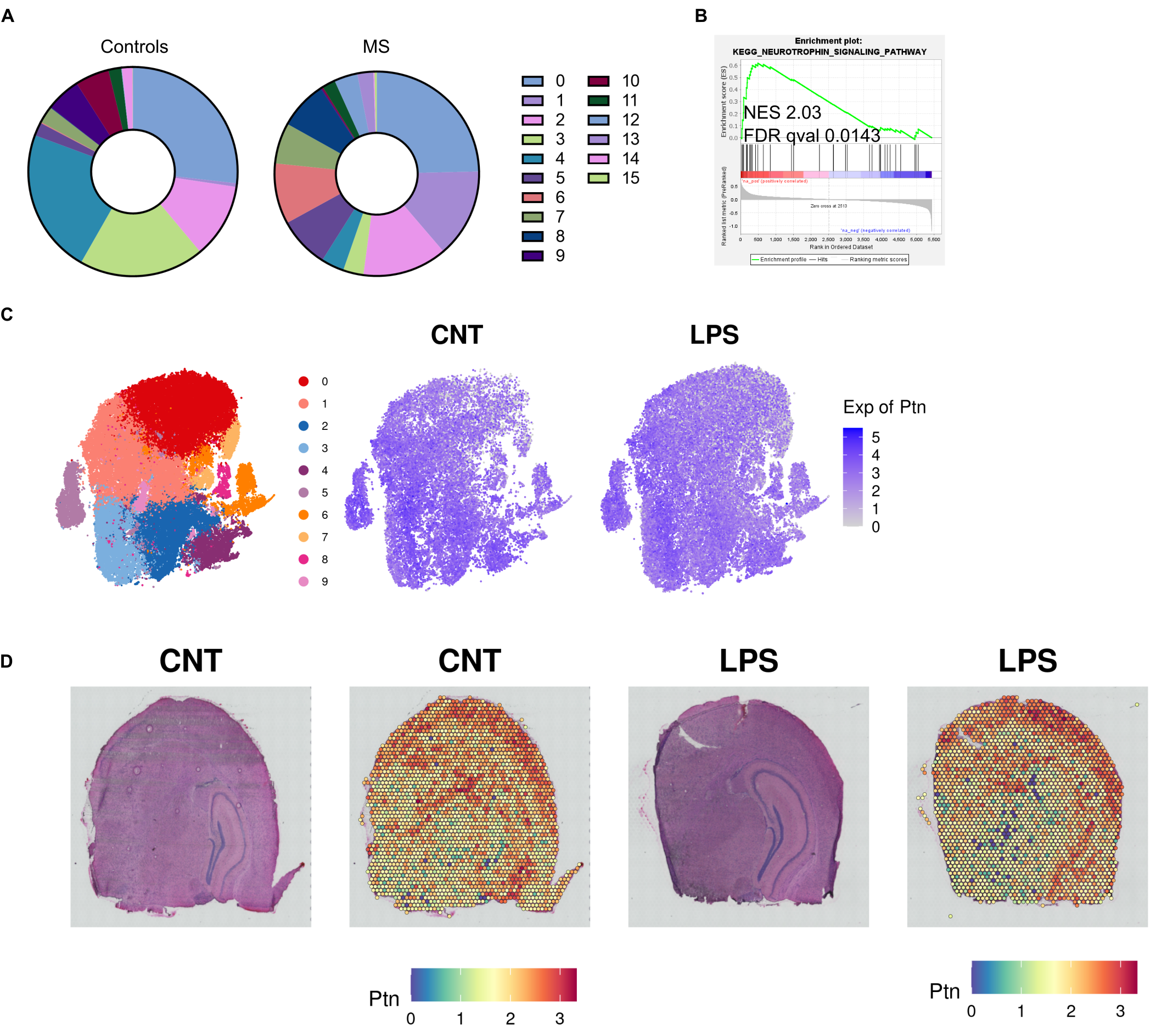

Supplement: Supplementary Figure 1 — (A) Abundance of astrocyte clusters as determined in Wheeler et al. (7) in control and MS patients. (B) Gene-set enrichment analysis of cluster 4 (KEGG hsa04722). (C) tSNE plot and Visium spatial expression (D) of Ptn expression in astrocyte subclusters following peripheral LPS- or vehicle (CONT)-injection obtained from Hasel et al. (4). [file Image_1.jpeg]

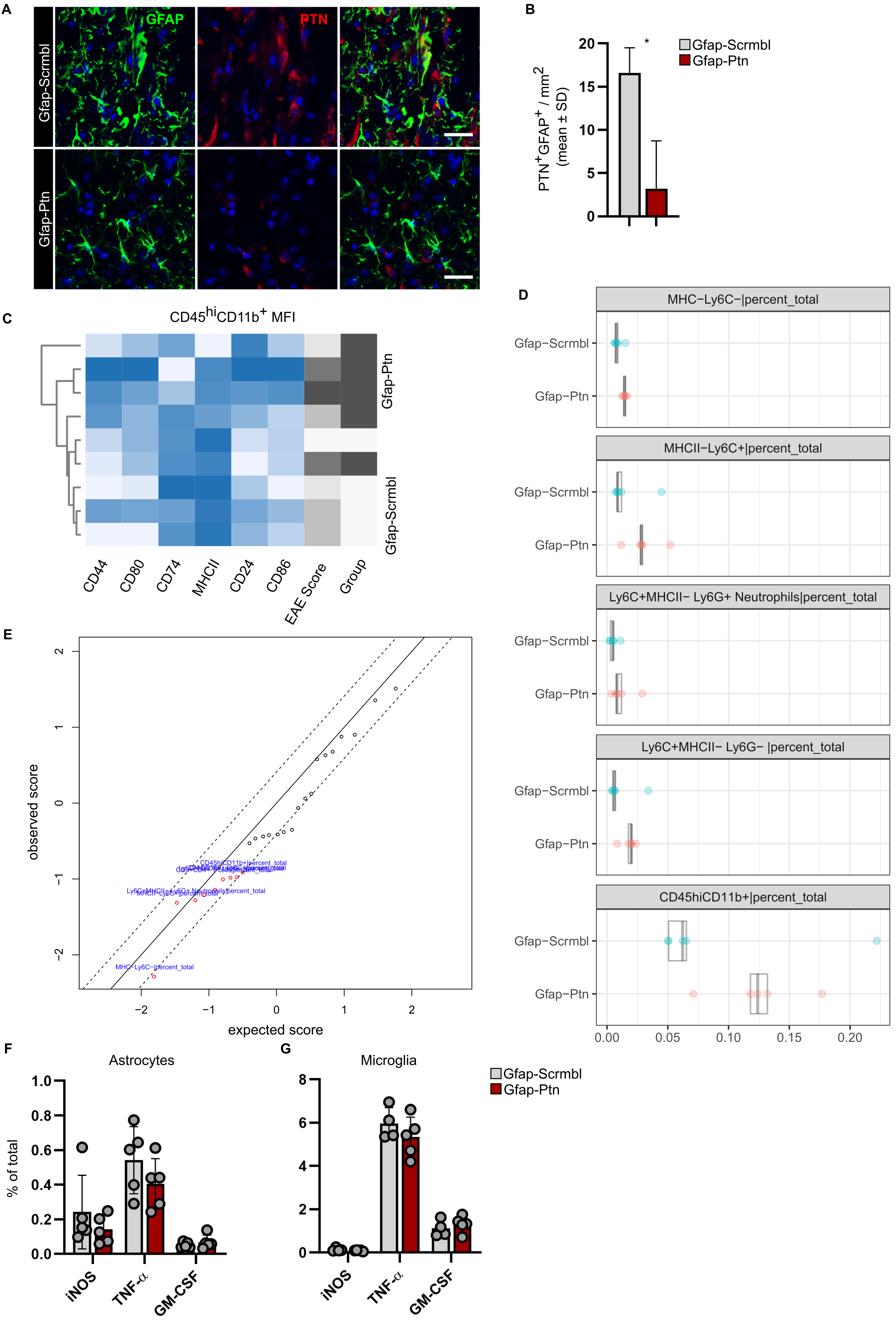

Supplement: Supplementary Figure 2 — (A) Immunostaining and quantification (B) of GFAP+PTN+ astrocytes in lumbar spinal cord pf Gfap-Scrmbl and Gfap-Ptn mice at day 28 post immunization. Scale bar represents 100 µm. Data shown as mean (n = 3) ± SD. Unpaired t test with Welch’s correction. * P < 0.05. (C) Median fluorescent intensities (MFIs) of activation markers expressed on CD45hiCD11b+ myeloid cells in CNS (brain and spinal cord) of Gfap-Scrmbl (n = 4) and Gfap-Ptn (n = 5) animals quantified by flow cytometry. (D, E) Significance Analysis of Microarrays (SAM) of cell populations in Gfap-Scrmbl (n = 4) and Gfap-Ptn (n = 5) mice at day 28 post immunization. (F) Cytokine production by astrocytes and microglia (G) in Gfap-Scrmbl (n = 4) and Gfap-Ptn (n = 5) mice at day 28 post immunization quantified by intracellular flow cytometry. Data shown as mean ± SD. [file Image_2.jpeg]

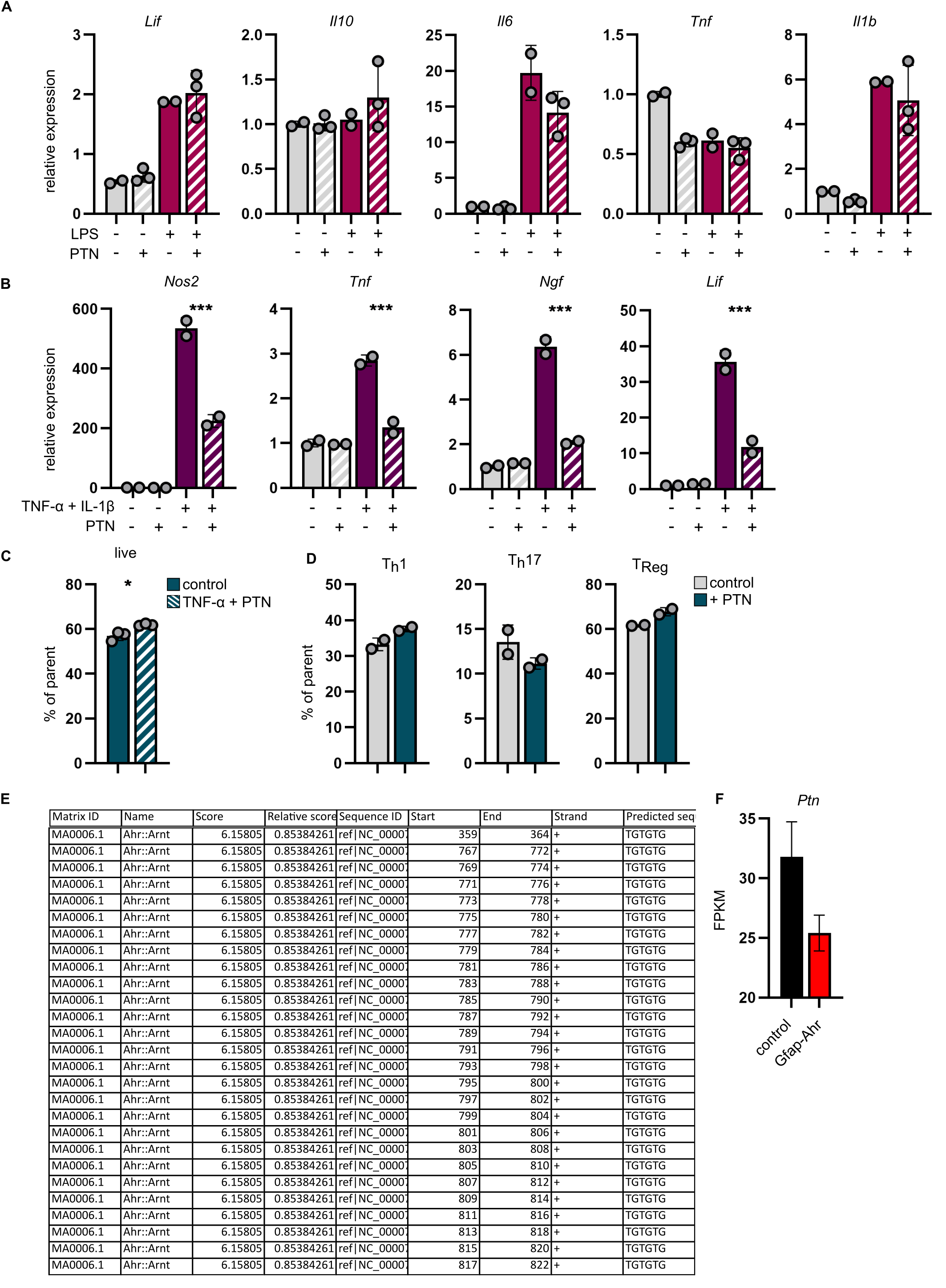

Supplement: Supplementary Figure 3 — (A) qPCR analysis of Ptn expression in unstimulated and LPS-stimulated microglia (A) and TNF-α/IL-1β-stimulated astrocytes (B) ± PTN. Data shown as mean ± SD. Unstim control n = 2, unstim + PTN n = 3, stim control n = 2, stim + PTN n = 3. Sidak’s multiple comparisons test. *** P < 0.001. (C) Quantification of live (Annexin V-PI-) N2A neuronal cells following stimulation with TNF-α ± PTN. Data shown as mean ± SD. Stim control n = 3, stim + PTN = 3. Unpaired t test with Welch’s correction. * P < 0.05. (D) Quantification of T cell subsets following differentiation ± PTN. Data shown as mean ± SD. Control n = 2, stim + PTN = 2. Unpaired t test with Welch’s correction. (E) Ahr : Arnt binding sites in the promoter regions upstream of the Ptn coding region, analyzed with JASPER (57). (F) FPKM values of Ptn expression in sorted astrocytes of control and Gfap-Ahr animals as obtained from Rothhammer et al. (27). [file Image_3.jpeg]

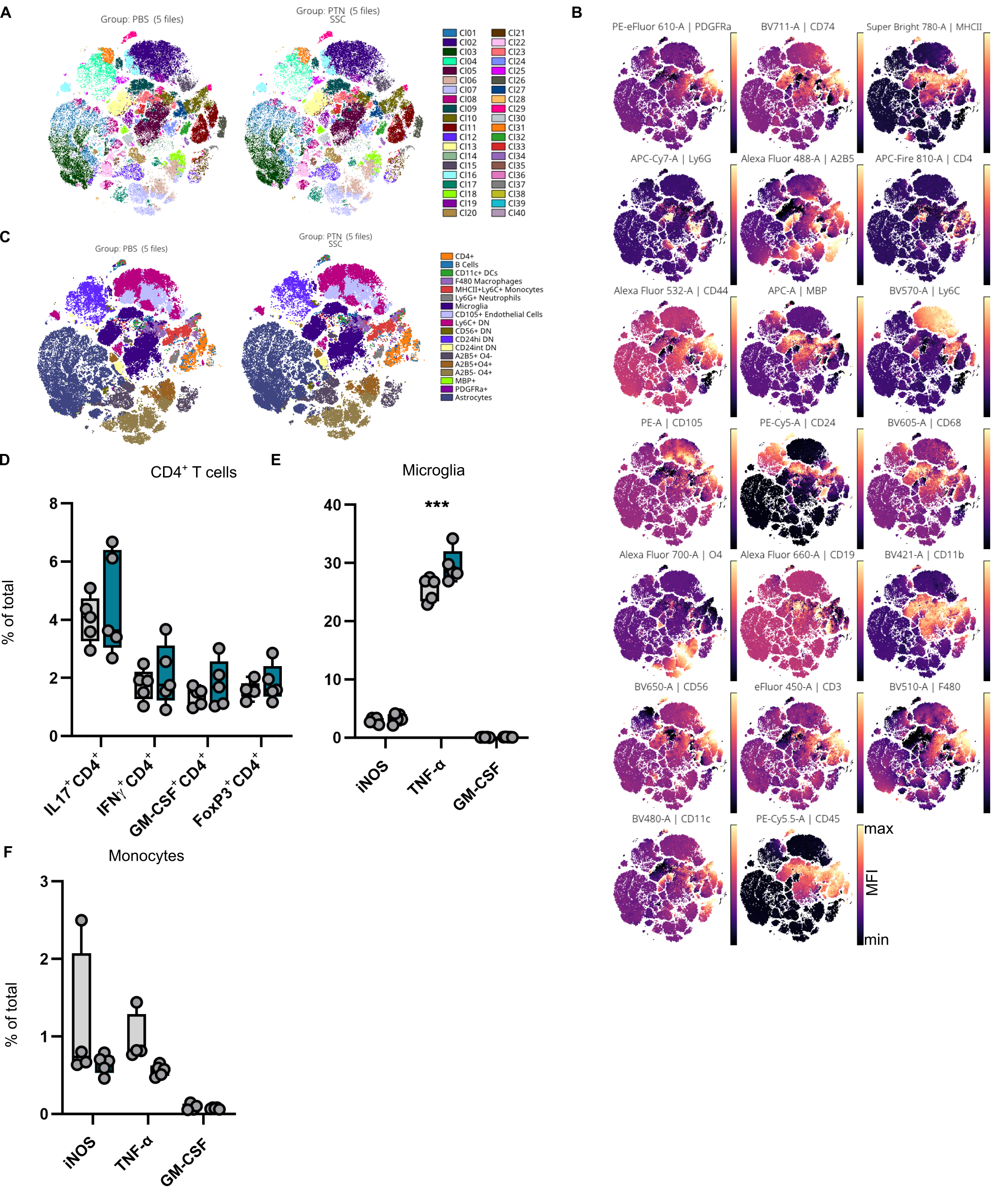

Supplement: Supplementary Figure 4 — (A) tSNE plot of CNS cells (brain and spinal cord) obtained from vehicle-treated (n = 5) or PTN-treated (n = 5) animals, quantified by high-parameter flow cytometry. Clusters were determined using PhenoGraph. (B) Marker expression (MFI) in respective clusters in pooled vehicle-treated and PTN-treated animals. (C) tSNE plot of CNS cells (brain and spinal cord) obtained from vehicle-treated (n = 5) or PTN-treated (n = 5) animals, quantified by high-parameter flow cytometry. Clusters were determined using manual gating. (D) Abundance of CD3+CD4+ T cell subsets in CNS (brain and spinal cord) of vehicle-treated (n = 5, grey), and PTN-treated (n = 5, blue) mice. (E) Cytokine production microglia and CD45hiCD11b+Ly6C+ monocytes (F) in CNS (brain and spinal cord) of vehicle-treated (n = 5, grey), and PTN-treated (n = 5, blue) mice. Sidak’s multiple comparisons test. *** P < 0.001. [file Image_4.jpeg]

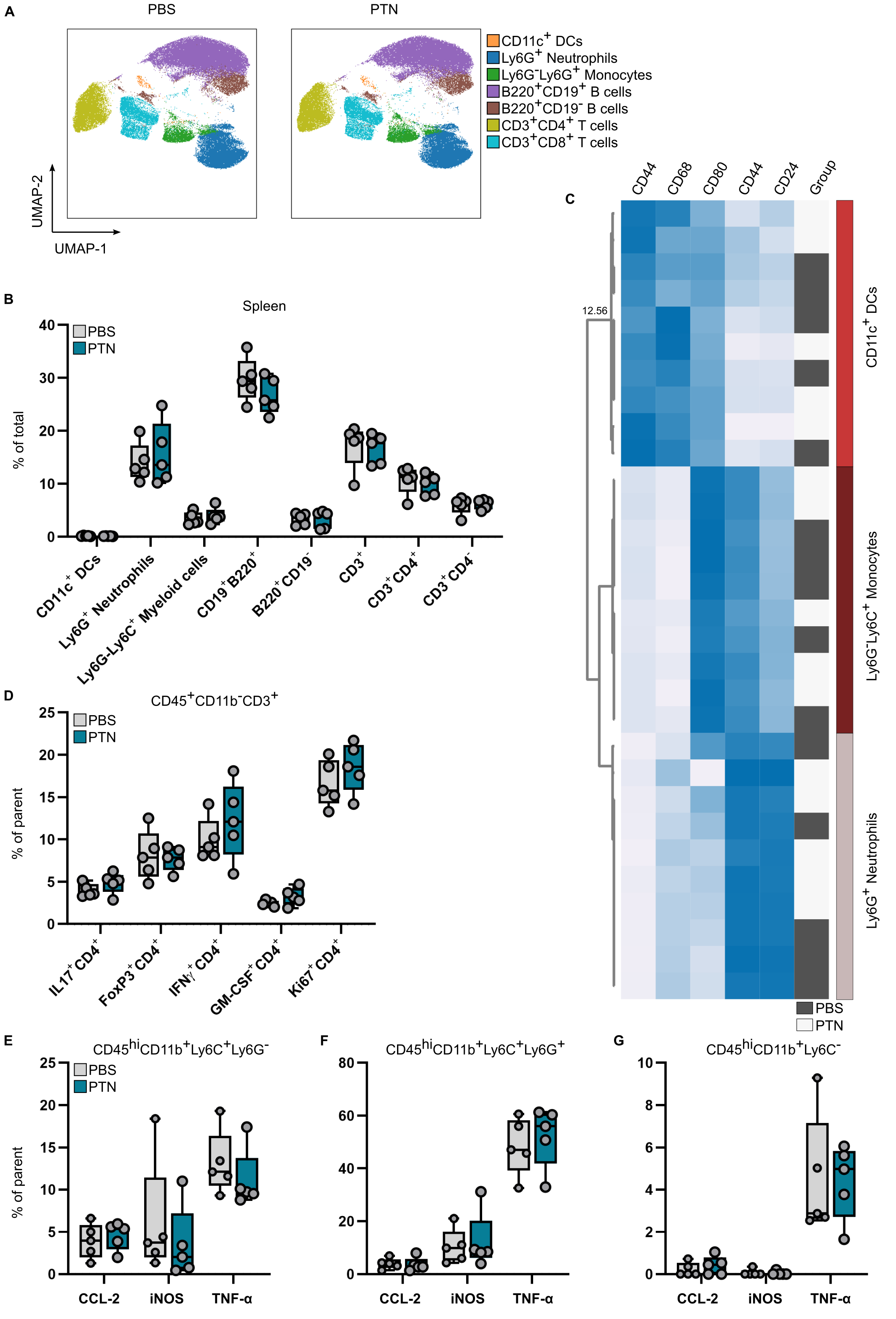

Supplement: Supplementary Figure 5 — (A) UMAP plot of splenic cells from vehicle-treated (n = 5) and PTN-treated (n = 5) mice at day 23 post immunization quantified by high-parameter flow cytometry. (B) Abundance of splenic cells from vehicle-treated (n = 5, grey) and PTN-treated (n = 5, blue) mice at day 23 post immunization quantified by high-parameter flow cytometry. (C) Median fluorescent intensities (MFIs) of activation markers expressed by CD11c+ dendritic cells, Ly6G- monocytes, and Ly6G+ neutrophils in spleens of vehicle-treated (n = 5, dark grey) and PTN-treated (n = 5, light grey) mice at day 23 post immunization. (D) Abundance of T cell subsets in spleens from vehicle-treated (n = 5, grey) and PTN-treated (n = 5, blue) mice at day 23 post immunization quantified by flow cytometry. (E) Quantification of cytokine production by Ly6G- monocytes, Ly6G+ Neutrophils (F), and CD45hiCD11b+Ly6C- myeloid cells (G) in spleens of vehicle-treated (n = 5, grey) and PTN-treated (n = 5, blue) mice at day 23 post immunization. [file Image_5.jpeg]

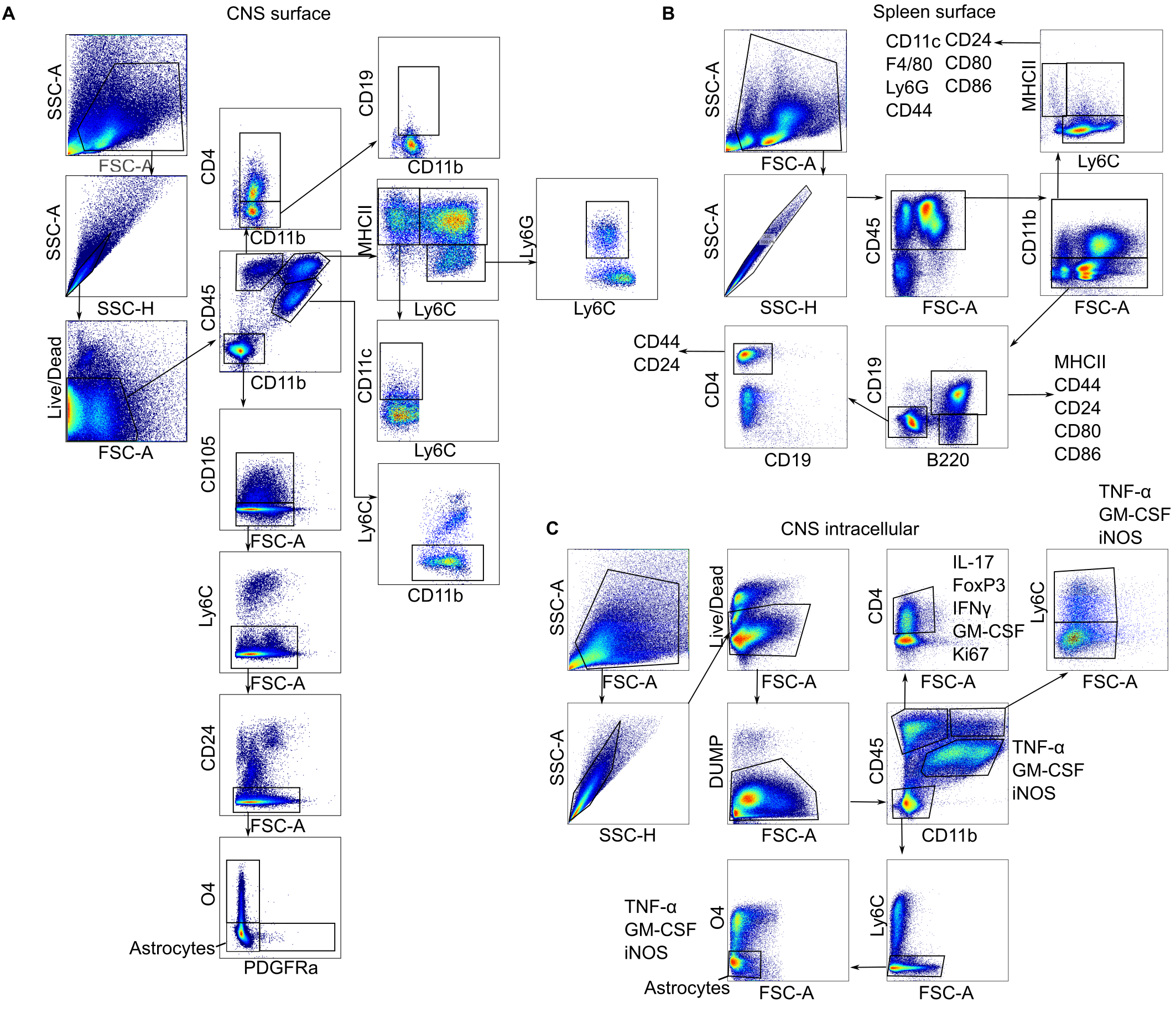

Supplement: Supplementary Figure 6 — (A) Gating strategy used for high-parameter flow cytometry surface staining of CNS cells. Astrocytes were identified by a negative gating strategy as described previously (27, 58, 59). (B) Gating strategy used for high-parameter flow cytometry surface staining of splenic cells. (C) Gating strategy used for intracellular staining of CNS cells. Following live/dead discrimination, a dump channel was used to exclude erythrocytes (TERR119), B cells (B220), OPCs (CD140a), Neutrophils (Ly6G). Astrocytes were identified by negative gating. For splenocyte intracellular staining, the same strategy was applied with the inclusion of CD19/B220. [file Image_6.jpeg]
